# Supplementary material for: Chromothripsis during telomere crisis is independent of NHEJ, and consistent with a replicative origin
Source: Genome Res. 2019 May;29(5):737–49. doi: 10.1101/gr.240705.118 (PMC6499312; doi:10.1101/gr.240705.118)
Supplement: Supplemental Material [file supp_gr.240705.118_Supplemental_file_1.zip › contigs/annotated_contigs/DB52/contig.2.DB52_length_407_mean_cov_3.31695331695.docx]

**DB52_length_407_mean_cov_3.31695331695**

```
TCAAAGAACTGAAAATGCAATTATCATTTGATCCGGCAATCCCACTACTGAGTATCTACACAAATGACAAGAAATCCACAATATCAAAA
 >chr13:54076418-54076562 - E=4e-72
AGATAACCTGTGTTTGTAAGCTAATCACAGCATTGTTCACAATAGCAAAGGTT|TG|TTCCCTGACCACTGTTATATCACATTAGGTGT
                                                      >chr13:54075676-54075941 - E=4e-1
AAATTATAAAATTTGAAAATATCTGCTTAGAATTTACTTATAGCTTTAAAAGAATTCTACTGTCTATGCCTTCTTAGAAGCTGGATATT
39
GGTGTCATTTTCTATCTTATGTGGTAAATCTCTAGGGGCAGTGGTAATGCTTATTTACATTACTTTTACTCACTACTTTTAACACTGAA

ATGTGAAACCAGGTCTCAAAAATAAATGTTTGTGATGACTCATTCCTCAGCCT
```
